# Developing an online food composition database for an Indigenous population in south-western Uganda

**DOI:** 10.1017/S1368980021001397

**Published:** 2021-06

**Authors:** Giulia Scarpa, Lea Berrang-Ford, Areej O Bawajeeh, Sabastian Twesigomwe, Paul Kakwangire, Remco Peters, Sarah Beer, Grace Williams, Carol Zavaleta-Cortijo, Didacus B Namanya, Shuaib Lwasa, Ester Nowembabazi, Charity Kesande, Holly Rippin, Janet E Cade

**Affiliations:** 1 School of Environment, University of Leeds, LS2 9JT, UK; 2 School of Food Science and Nutrition, University of Leeds, UK; 3 Department of Food and Nutrition, Faculty of Human Sciences and Design, King Abdulaziz University, Jeddah, Saudi Arabia; 4 Indigenous Health Adaptation to Climate Change Research Team, Kanungu District, Uganda; 5 Leeds Institute of Health Sciences, University of Leeds, UK; 6 Dietary Assessment Ltd, UK; 7 Facultad de Salud Publica y Administracion, Universidad Peruana Cayetano Heredia, Peru; 8 Ministry of Health, Uganda; 9 Department of Geography, Makerere University, Kampala, Uganda; 10 The Global Center on Adaptation, Rotterdam, Netherlands; 11 WHO European Office for Prevention and Control of Non-communicable Diseases (NCD Office), Moscow, Russian Federation

**Keywords:** Food composition databases, Indigenous population, South-western Uganda, Nutritional assessment, Online food database

## Abstract

**Objective:**

To develop an online food composition database of locally consumed foods among an Indigenous population in south-western Uganda.

**Design:**

Using a community-based approach and collaboration with local nutritionists, we collected a list of foods for inclusion in the database through focus group discussions, an individual dietary survey and markets and shops assessment. The food database was then created using seven steps: identification of foods for inclusion in the database; initial data cleaning and removal of duplicate items; linkage of foods to existing generic food composition tables; mapping and calculation of the nutrient content of recipes and foods; allocating portion sizes and accompanying foods; quality checks with local and international nutritionists; and translation into relevant local languages.

**Setting:**

Kanungu District, south-western Uganda.

**Participants:**

Seventy-four participants, 36 Indigenous Batwa and 38 Bakiga, were randomly selected and interviewed to inform the development of a food list prior the construction of the food database.

**Results:**

We developed an online food database for south-western Uganda including 148 commonly consumed foods complete with values for 120 micronutrients and macronutrients. This was for use with the online dietary assessment tool myfood24. Of the locally reported foods included, 56 % (*n* 82 items) of the items were already available in the myfood24 database, while 25 % (*n* 37 items) were found in existing Ugandan and Tanzanian food databases, 18 % (*n* 27 items) came from generated recipes and 1 % (*n* 2 items) from food packaging labels.

**Conclusion:**

Locally relevant food databases are sparse for African Indigenous communities. Here, we created a tool that can be used for assessing food intake and for tracking undernutrition among the communities living in Kanungu District. This will help to develop locally relevant food and nutrition policies.

Food composition database information with relevant local foods is required to assess nutritional intake. Indeed, evidence-based nutrition policies needed to improve population nutrition-related health may be lacking without the analysis of individual energetic and nutrient intake^(1)^. However, few countries hold accurate and up-to-date food composition tables. Many low- and middle-income regions lack these data and use other countries’ food composition tables as a proxy^(2)^.

To investigate population food intakes, methods include 24-h recall studies, food diaries and FFQ, though all methods are prone to substantial measurement error^(3)^. The 24-h recall is the most common nutritional assessment method in low- and middle-income countries^(3)^. It is frequently used to obtain energetic and nutritional intake measurements and assess dietary quality in individuals and populations across a range of cultural contexts^(4–5)^. Nowadays, electronic, automated systems have been validated and used to reduce costs and time of data collection^(6–12)^.

All methods to collect food intake data must be underpinned by food composition databases appropriate for the study population. However, those are often inadequate, especially in highly food-insecure contexts where the type and quantity of ingredients in a recipe can change depending on food availability (in submission). Engaging with the community and local nutritionists can be the key to getting more accurate food data and to understanding the evolution of populations’ dietary patterns overtime. Such information can also enrich the level of details that we need to develop relevant food composition tables in low-income and food-insecure settings (in submission).

Food composition databases representing food consumed by Indigenous communities are scarce. We contribute to this knowledge gap by constructing an online database of locally consumed foods with complete nutrient information for two vulnerable populations in south-western Uganda, the Batwa and Bakiga. Like other low-income countries, no food composition tables are available for south-western Uganda. The only existing food database was designed for central and eastern Uganda^(13)^ and does not include all common recipes and local foods eaten by the Batwa and Bakiga communities (Scarpa *et al.*, 2020, in submission).

We created a framework to collect unique and relevant food and recipes data for a specific population by engaging with the local community, and we formulated the following steps to develop the food composition database: (1) to identify foods for inclusion in the database; (2) to clean initial data and remove duplicate items; (3) to link foods to existing food composition tables; (4) to map and calculate nutrient content of recipes and foods; (5)to allocate portion sizes and accompanying foods; (6) to perform quality checks with local and international nutritionists and (7) to translate food items into relevant local languages.

## Methods

### Study region and population

The Batwa community, self-identified as Indigenous, lives in south-western Uganda and represents a minority group in Kanungu District^(14)^. The Bakiga community resides in the same area and constitutes the majority population in the district^(15,16)^. The Batwa were originally hunter-gathers until their displacement in 1991 from the forest, now known as Bwindi Impenetrable National Park^(17)^. They became a sedentary population frequently employed by the Bakiga in agriculture. However, many Batwa still rely on humanitarian aid to survive^(18)^. Conversely, the Bakiga are traditionally farmers whose income comes primarily from food crops^(16)^. Both populations are highly vulnerable to food insecurity, malnutrition (especially stunting), acute gastrointestinal diseases (including vomiting, diarrhoea and associated symptoms) and malaria^(19)^.

### Study design

**Fig. 1:**
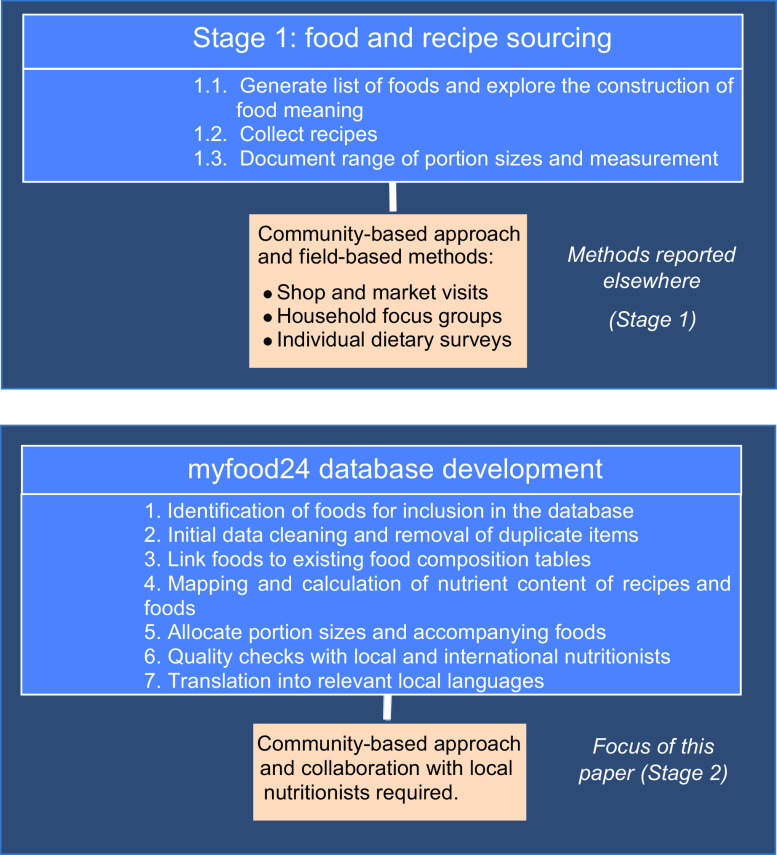
Framework used to characterise the south-western Ugandan food database. This paper documents the methods and results from Stage 2

The food database construction for south-western Uganda follows the framework in Fig. 1. The framework aimed at constructing food composition tables for low-income areas. It is composed of two stages: the first stage comprised fieldwork and a mixed methodology to collect a list of foods consumed, along with recipes and portion sizes (Scarpa *et al.*, 2020, in submission). To develop the online south-western Ugandan database, we used six steps for food composition database creation previously used by other researchers^(6,20)^. The data on foods and recipes were collected by the local research team, and the food database was created by the PI (GS) in collaboration with local nutritionists and myfood24 team in UK.

### Stage 1

Detailed methods and results from Stage 1 are presented elsewhere (Scarpa *et al.*, 2020, in submission), with this paper focusing primarily on Stage 2. In the first stage, food and recipe data were collected through market and shop assessment (*n* 4 markets and 10 shops visited in total), focus group discussions (*n* 8 in total) and individual dietary survey (*n* 76) from July to October 2019 (see online Supplemental material, Appendix 1). A male local researcher supported by a Batwa and a Bakiga female researcher conducted the fieldwork. The data were collected in the local Rukiga language and then translated in English by the local researcher (Scarpa *et al.*, 2020, in submission).

For the individual dietary survey and focus groups (74 participants in total, 36 Batwa and 38 Bakiga), four settlements among Batwa and four adjacent (geographically matched) Bakiga settlements were sampled, ensuring a representative sample of people of different age and sex. To explore the range of foods in different geographical areas, data were collected in four different Batwa and Bakiga communities: close to the markets and shops, far from the markets and shops, mid-way between the two zones and close to the forest. Individuals participating in the study were randomly selected from village lists (Scarpa *et al.*, 2020, in submission)^(3)^.

### Stage 2

Stage 2 was comprised seven steps summarised in Fig. 1.

### Identification of foods for inclusion in the database

We identified the most common foods and dishes consumed among the Batwa and Bakiga communities and we removed non-edible items (e.g. cigarettes). Additionally, a food photo album briefing was created with the pictures of local foods taken by a local nutritionist. It consisted of a description of the most frequently eaten foods and local recipes, and associated images of tools used to measure portion sizes. This supported the development of the food database by giving background information to non-local nutritionists on traditional foods consumed by the Batwa and Bakiga.

### Initial data cleaning and removal of duplicate items

We cleaned the data and removed duplicates. We included all different cooking methods and ingredients for each food and dish eaten in south-western Uganda. Unit conversions were computed to match myfood24 database values; for example, some micronutrients were converted from grams to milligrams. Additionally, some missing values such as kilojoules were calculated using mathematical formulae (e.g. we used the value of the kilocalories and we divided it by 4·184).

### Linking foods to existing food composition tables

Four existing food composition tables^(13,21–23)^ from Uganda, Kenya, Tanzania and the UK were identified as relevant for the creation of the local food table.

The Ugandan database was created in 2012, and it includes 728 foods and recipes. No foods were chemically analysed for nutrients in the Ugandan table, except for orange sweet potato^(13)^. Most of the food items were sourced from the USDA^(24)^ food composition table. The Tanzanian and the Kenyan food composition tables^(21)^ and myfood24 database were consulted if a dish or item was not available in the Ugandan food database. The Tanzanian food composition database was developed in 2008 and comprises over 400 foods and recipes, while the Kenyan food composition table was created in 2018 and contains 509 foods^(22)^. The myfood24 database includes UK, German, Danish, and Australian foods and recipes, both branded and generic products. The data on nutritional content in the British food table come from the UK Composition of Foods Integrated Database (COFID)^(23)^ for generic products and back-of-pack nutrition labels for branded items which are mapped to similar generic products in COFID to allocate nutrient values for the remaining nutrients.

We used back-of-pack labels of packaged foods identified in Uganda to collect nutritional content information when this was not available in existing food composition tables. The estimated portion sizes were derived from focus group discussions which we held in the first stage of this study.

### Mapping and calculation of nutrient content of foods and recipes

Recipes were collected during the Stage 1 of the study. In the case of beans-based dishes, two versions of the same recipe, one with fresh and the other with dried beans, were used, as both were cooked and eaten by the communities in similar amounts.

When possible, we matched the south-western Ugandan foods with the items already available in the myfood24 database (Table 1). This database is more complete in terms of nutritional content analysed than the other food composition tables. It contains 120 nutrients available for each food item^(6)^. The decision to keep a food item already available in myfood24 was made by three independent researchers, including at least one Ugandan nutritionist.

**Table 1:** Example of matched foods for creating the online south-western Ugandan myfood24 database

Ingredients and local dishes in Rukiga language with English translation in brackets	Closest matched foods
Emboga zadodo (dodo)	100 % boiled amaranth leaves
Akatogo kebitookye nebihimba byomiire (matoke with dried beans)	70 % banana (matoke), 1 % salt, 29 % dried beans (average of three different types of beans: blackeye beans, haricot beans and red kidney beans)
Esano yoburo egoyire/oburo (millet bread)	100 % boiled millet flour
Emboga zadodo nebihimba byomir (dodo with dried beans)	31 % amaranth leaves, 1 % salt, 68 % dried beans (average of three different type of beans: blackeye beans, haricot beans and red kidney beans)

Cooked ingredients were used for nutrient information for the majority of the recipes. Nutritional and energetic content was available from existing African food databases. Therefore, we did not add water nor calculate the cooking factor as those elements were already included in the cooked food items. Only in the case of two dishes, firstly we converted the raw ingredients of the recipes to percentages, to facilitate allocation of nutrients to each composite food per 100 g^(6)^. Secondly, we applied cooking factors to calculate the quantity of cooked items^(18)^. Lastly, we calculated water evaporation or absorption as required^(1)^.

To determine the ingredients’ quantity in the dishes, we used an average of each ingredient described by the participants for every recipe; then, we created nutrient values for each recipe using the percentage of each ingredient in the recipe.

### Allocate portion sizes and accompanying foods

Each food and dish was associated with portion size measures (Scarpa *et al.*, 2020, in submission). For example, for quantifying boiled dried beans, we included in the database the portions expressed in grams and in soup spoons.

Additionally, we added a list of ‘accompanying foods’ which automatically appears on myfood24 when a food item that is commonly consumed with another one is selected. For example, milk and sugar were identified as ‘accompanying ingredients’ for ‘porridge’.

### Quality checks with local and international nutritionists

A team composed of two nutritionists in Uganda and two nutritional researchers in the UK conducted quality checks after the first stage of fieldwork. Subsequently, two independent researchers and the myfood24 team checked the calculation of the recipes and the mapping of foods to ensure nutrient values were sensible.

### Translation into relevant local languages

Translation of food names was performed by a local nutritionist and checked by the second Rukiga speaker. Synonyms of foods were added to facilitate searching in the database.

### Data analysis

The data were analysed using Excel® for calculating the recipe ingredients and for basic descriptive statistics. MS Access® was used to create a database for inclusion into myfood24. This included all foods and nutrients, along with standard portion size information, prompts for foods commonly consumed with dishes and mispellings or different names to indicate foods if necessary.

## Results

### South-western Ugandan foods and dishes available in myfood24 database

The south-western Ugandan food database contains 148 of the most consumed foods among the Batwa and Bakiga communities living in Kanungu District, with complete information on 120 micronutrients and macronutrients. The foods were collected through the focus group discussions (collected information on 139 foods), individual dietary surveys (collected information on 69 foods), and shops and markets assessment (collected information on 95 foods). Some foods identified by the communities through one method (e.g. focus group discussions) were repeated in another method (e.g. market and shop assessments or individual dietary survey). We identified 303 food items using the three methods, representing 148 unique items after duplicate removal.

**Fig. 2:**
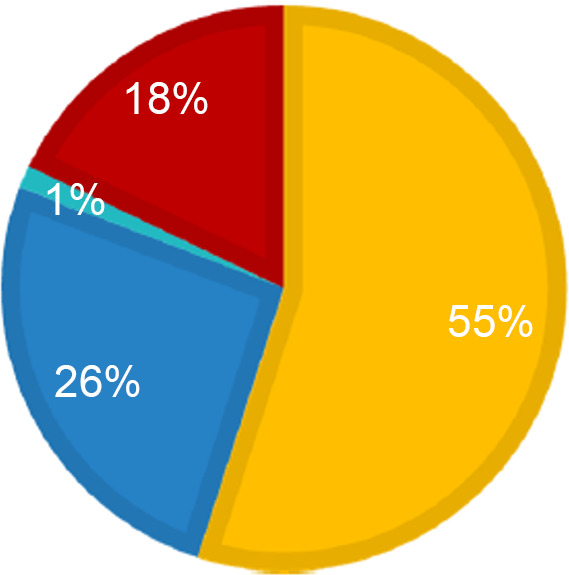
Sources used to develop the south-western Uganda myfood24 database (*n* 148 items). 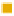, myfood24; 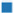, African food composition tables; 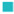, food labels; 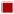, generated recipes

As shown in Fig. 2, the sources for developing the south-western Ugandan food composition database were multiple. Fifty-six per cent (82 items) of the foods were already available in the myfood24 database from the generic UK COFID tables, while the other 43 % (64 items) were added from African food composition tables (26 %) and generated recipes (18 %). Only 1 % (2 items) of the nutrients in foods included in the composition database were extracted from back-of-pack labels.

The south-western Ugandan myfood24 database included 43 % (64 of 148) of fruit and vegetable items and dishes, 26 % (38 of 148) of cereals items and cereal-based dishes (including dishes with higher percentages of cereals than vegetables), 14 % (20 of 148) of meat and fish dishes, and 5 % (7 of 148) of eggs and diary. Only 3·5 % (5 of 148) of products were sugary or sweet-based and 3 % (5 of 148) were included in oils, fats and condiments (Table 2). Some cooking oils and fats were branded, while the majority of the other food items did not have any brand. Soft drinks (4 %, i.e. 6 of 148) and alcoholic beverages (1·5 %, i.e. 2 of 148) corresponded to 4·5 % of the foods included in the database.

**Table 2:** Sources of the food list and the food database (myfood24 database, African food composition tables, back-of-pack labels products and generated recipes)

		Food list sources*			Food database sources			
Food category	Total (*n* 148 foods)	Focus groups	Individual dietary assessment	Market and shops assessment	Food already in myfood24 (*n* 82)	African food composi-tion tables (*n* 37)	Back-of-pack labels products (BOP) (*n* 2)	Generated recipes (*n* 27)
Fruits and vegetables	53	50	31	48	40	13	0	0
Meat and poultry	11	9	0	5	8	3	0	0
Dairy and eggs	7	7	2	6	7	0	0	0
Cereals and tubers	18	17	7	10	11	7	0	0
Drinks	6	6	3	6	4	0	2	0
Honey and other sugars	4	4	1	3	2	2	0	0
Fish	9	8	1	6	3	6	0	0
Oils and fats	3	3	2	3	3	0	0	0
Sauces and condiments	2	2	2	2	2	0	0	0
Alcohol	2	2	2	0	1	1	0	0
Cakes, biscuits, chocolates and other snacks	1	1	0	1	1	0	0	0
Dishes with vegetables and cereals or tubers	15	15	10	0	0	0	0	15
Dishes with fish and vegetables	1	1	0	0	0	0	0	1
Dishes cereal-based	5	5	1	5	0	5	0	0
Dishes vegetable-based	11	9	7	0	0	0	0	11

* The total of foods collected through focus group discussions, individual dietary surveys and shop and market assessments is not equal to 147, but it is higher. However, some foods and recipes were repeated using different methods.

The composite foods (including recipes already available in other food composition tables and new generated recipes) were mostly bean- and vegetable-based (9 of 32) or bean- and cereal-based (10 of 32) (Table 2). Thirty-one of thirty-two composite foods did not contain any animal protein. We inserted in our composition database five recipes (5 of 32) that were already available in the Ugandan food composition table. A selection of nutrients calculated for the twenty-seven generated recipes is reported in Table 3 and shown as per 100g of the cooked dish (the complete information can be found in Supplementary material-Appendix 2). The food products used for cooking were fresh or dried, and no recipes for preparing soups were mentioned. A high proportion of foods that needed to be cooked before consumption were boiled, except for some items such as fish and meat which were either boiled or fried. Sauces and condiments were prepared at home, while biscuits and sweets were packaged and bought from the markets and shops.

**Table 3:** Description of south-western Ugandan generated recipes (for 100 g of each cooked dish)

Local dishes	Nutrients (for 100 g of each cooked dish)									
	Energy (Kcal)	CHO* (g)	Fat (g)	Prot† (g)	Ca‡ (mg)	Fe§ (mg)	Vit|| C (mg)	Zn¶ (mg)	Vit** A (mcg)	Folate (µg)
Boiled matoke with boiled fresh beans†† (70 % matoke, 29 % beans and 1 % salt)	82	19·3	0·3	2·4	11·0	0·8	4·6	0·3	1·4	25·7
Boiled matoke with boiled dried beans‡‡ (70 % matoke, 29 % beans and 1 % salt)	89	20·3	0·4	3·0	14·8	0·9	4·8	0·5	1·7	32·1
Boiled dried beans with boiled yellow sweet potatoes (70 % yellow sweet potatoes, 29 % beans and 1 % salt)	110	24	0·2	3·7	38·6	1·2	1·7	0·6	124·1	33·4
Boiled dried beans with boiled orange sweet potatoes (70 % orange sweet potatoes, 29 % beans and 1 % salt)	110	24	0·2	3·7	40·0	1·2	1·8	0·6	306·1	33·4
Boiled fresh beans with boiled yellow sweet potatoes (70 % yellow sweet potatoes, 29 % beans and 1 % salt)	103	23	0·1	3·1	34·8	1·1	1·9	0·5	123·9	27·1
Boiled fresh beans with boiled orange sweet potatoes (70 % orange sweet potatoes, 29 % beans and 1 % salt)	103	23	0·1	3·1	36·2	1·1	2·0	0·5	305·9	27·1
Boiled dried beans with boiled yam (70 % yam, 29 % beans and 1 % salt)	124	28·4	0·4	3·5	20·4	1·0	2·9	0·6	0·3	28·6
Boiled fresh beans with boiled yam (70 % yam, 29 % beans and 1 % salt)	117	27·3	0·3	2·8	16·7	0·9	3·0	0·5	0·0	22·1
Boiled pumpkin and boiled dried beans (70 % pumpkin, 29 % beans and 1 % salt)	44	8·7	0·2	2·8	22·5	1·1	3·4	0·4	175·3	30·7
Boiled green leaves§§ with boiled fresh beans (79 % beans, 20 % green leaves and 1 % salt)	69	12·3	0·2	5·0	50·6	1·8	3·3	0·6	28·8	57·8
Boiled green leaves with boiled dried beans (79 % beans, 20 % green leaves and 1 % salt)	87	5·5	0·5	6·7	60·9	2·1	3·3	0·8	29·6	75·2
Boiled fresh beans with boiled dodo (68 % amaranth leaves, 31 % beans and 1 % salt)	60	10	0·2	4·8	87·3	2·1	10·2	0·6	76·0	58·0
Boiled dodo and groundnuts (68 % groundnuts, 31 % amaranth leaves and 1 % salt)	221	14·6	15·0	10·1	105·7	1·4	9·8	1·4	75·9	66·8
Boiled peas with boiled matoke (70 % matoke, 29 % peas and 1 % salt)	82	17·9	0·7	3·4	8·4	0·6	9·1	0·4	13·6	15·6
Boiled peas and orange sweet potatoes (70 % orange sweet potatoes, 29 % peas and 1 % salt)	103	21·6	0·5	3·4	33·6	1·0	6·4	0·6	318·1	16·9
Boiled peas and yellow sweet potatoes (70 % yellow sweet potatoes, 29 % peas and 1 % salt)	103	21·6	0·5	3·4	32·2	1·0	6·3	0·6	136·1	16·9
Fried fish|||| with groundnuts sauce (38 % fish, 13 % onion, 32 % groundnuts paste, 16 % tomatoes and 1 % salt)	356	8·2	19·2	41·3	35·9	1·7	2·5	1·5	5·3	59·4
Boiled fresh beans with groundnuts sauce (34 % fresh beans, 14 % onion, 34 % groundnuts paste, 17 % tomatoes and 1 % salt)	236	13·6	17·3	10·7	29·1	1·4	2·9	1·3	5·7	49·9
Boiled fresh beans with dried maize (33 % fresh beans, 66 % maize grain and 1 % salt)	151	30·1	1·7	5·1	12·0	1·5	0·3	1·0	0·0	26·4
Boiled fresh beans and Irish potatoes (70 % Irish potatoes, 29 % peas and 1 % salt)	74	16·1	0·1	2·9	11·7	0·9	4·4	0·4	0·0	31·2
Boiled cassava and peas (70 % cassava, 29 % peas and 1 % salt)	114	26·3	0·6	2·3	17·5	0·8	16·6	0·5	12·2	15·5
Boiled pumpkin and fresh beans (70 % pumpkin, 29 % beans and 1 % salt)	37	7·6	0·1	2·1	18·7	1	3·5	0·3	175·0	24·2
Boiled dried beans and Irish potatoes (70 % Irish potatoes, 29 % peas and 1 % salt)	81	17·2	0·2	3·6	15·5	1·0	4·3	0·5	0·3	37·7
Boiled peas and Irish potatoes (70 % Irish potatoes, 29 % peas and 1 % salt)	73	14·8	0·5	3·2	9·1	0·7	8·8	0·5	12·2	21·1
Boiled dodo with boiled dried beans (68 % amaranth leaves, 31 % beans and 1 % salt)	76	12·4	0·5	6·3	96·2	2·3	9·8	0·9	76·6	72·9
Boiled mushroom and eggplants (66 % eggplants, 33 % mushrooms and 1 % salt)	25	5·5	0·3	1·4	8	0·7	2·7	0·4	0·7	20·5

* Carbohydrates.

† Proteins.

‡ Ca.

§ Fe.

|| Vitamin C.

¶ Zn.

** Vitamin A.

†† Fresh beans: fresh kidney beans.

‡‡ Dried beans: mix of dried blackeye beans, dried haricot beans and dried red kidney beans.

§§ Green leaves: different green leaves, including amaranth leaves, eggplants leaves and cabbage leaves.

|||| Fish: tilapia.

Many identified generic fruit and vegetable items were already available in the myfood24 database (45 of 58 items). The new vegetables items added to the online database contained various types of wild leafy vegetable and fruit (thirteen new items).

Local fish and meat, commonly consumed in south-western Uganda, were also added to the database (e.g. goat meat). Their nutritional content was derived from the Ugandan and UK food composition tables.

The identified branded products had incomplete nutrient content information on the back-of-pack label and therefore had to be matched to similar foods from the myfood24 database which held complete nutritional data. The products, reported in Table 4, are mostly produced in Uganda or in Africa, and one is an international item.

**Table 4:** Branded products missing some important nutritional information that required mapping of nutrients

Branded product	Type of food	Country of production	Matched with
Waragi	Alcohol	Uganda	100 % gin
Blue Band	Cooking fat	International	100 % margarine
Coke, Fanta	Drinks	Uganda	Already in myfood24 database
Kimbo	Cooking fat	Africa	100 % margarine
Butto	Cooking oil	Uganda	100 % vegetable oil
Kayonza	Tea	Uganda	100 % black tea

## Discussion

We created an online food database with the most common foods and dishes consumed by the Batwa and Bakiga communities. Recognising the importance of collecting information on food meanings and social and environmental factors that affect individual food consumption, we used multiple fieldwork techniques and a community-based approach (Scarpa *et al.*, 2020, in submission). We also believe that gaining knowledge on communities’ food systems can increase the understanding of local nutrition practices and facilitate the collection and analysis of local food intake data. Designing a locally relevant food composition database may be useful for monitoring the prevalence of undernutrition and identifying individuals potentially at risk of nutritional deficiencies (e.g. Fe)^(25)^. It can also inform nutrition policies and future health intervention towards the third Sustainable Development Goal, ‘Zero Hunger’^(26)^.

### Challenges in developing the south-western composition database

We used different food composition tables to calculate the nutritional content of foods such as the Ugandan food composition database containing data from USDA (United States Department of Agriculture) and UK food composition tables. As a result, the nutrient content of foods analysed in high-income settings may not fully represent the nutritional values of an Indigenous meal as those vary according to the geographical position, but also food colour, species and season^(1,27–30)^. Therefore, relying on Western food analysis may lead to miscalculation of the real nutritional content^(31)^. Although we are aware of possible limitations, before inserting any food in the myfood24 database, the Ugandan and UK teams evaluated the existing nutritional content. We thus included the most accurate nutritional values for each food according to existing knowledge. Indeed, we compared the macronutrient and micronutrient content in four databases (African and UK myfood24 databases), and if the nutritional values of African food composition tables were similar to those in myfood24 database, we choose to insert myfood24 values as they contained detailed analysis of over 120 nutrients.

Data on food labels were missing as in many low-income countries^(32–35)^; therefore, we could not extract information on nutrients except for two food items. In addition, we could not include some leafy vegetables consumed by the communities to cure specific diseases, due to lack of information on nutritional content in the scientific literature. We recommend further research to analyse the nutritional value of local plants and traditional herbs to be inserted in the south-western Ugandan food database. Including local foods also underpins a focus on Indigenous justice and the importance of recognising and valuing Indigenous food systems and practices, which in many contexts have been found to have nutritional and environmental benefits for communities^(36)^.

Portion sizes were also challenging to measure as the Batwa and Bakiga often ate from the same plate. The participants in the focus group discussions did not like the potential to illustrate portions of dishes consumed through pictures^(37)^, a medium often used in high-income countries to support food portion size estimation^(38,39)^. Household measurements were used to quantify food portions not only for the composition database of south-western Uganda but also for other African and South-Asian countries such as Burkina Faso and Vietnam^(39)^. A more precise method may be needed to assess individual portion sizes. The use of wearable cameras was investigated by Bulungu *et al.* ^(40)^ in Eastern Uganda to explore type of foods consumed and nutrition practices; however, further research is required to assess the reliability of this method to measure food portions in extremely low-income contexts.

### Recipes and foods low in energies and poor in nutrients

As in other lower-income countries, economic constraints caused low consumption of foods rich in proteins and fats in south-western Ugandan populations^(41)^. In previous focus group discussions (Scarpa *et al.*, 2020, in submission), Batwa and Bakiga participants argued that in the past, when they lived in the forest, they used to hunt and often ate wild meat. Evidence suggests that hunting saved from starvation of different Indigenous communities, for example, the Inuit in the Arctic^(42)^. However, the study participants reported that now their diet is low in animal proteins, and the consumption of vegetable- and beans-based dishes is higher due to the high cost of meat in the markets.

The energetic level of the Batwa and Bakiga dishes is, in fact, generally low, and some micronutrients including Fe content may not be adequate to satisfy nutritional requirements, particularly of children and pregnant women. In vegetable protein-based dishes, non-haem Fe absorption varies between 5 % and 12 %. The variation depends on individual Fe status and other factors such as food preparation and Ca content^(43–45)^. Phytate and other inositol phosphates, which are present in many edible plants, may also inhibit Fe absorption^(46–48)^. Conversely, if the Fe intake comes from mixed diets (animal and vegetable-based dishes), the quantity of Fe absorbed is between 14 % and 18 %, and the influence of inhibitors of Fe absorption is reduced, with a smaller effect on individual’s Fe status^(43,44)^.

Most of the local dishes collected had a total of kilocalories between 25 and 236 per 100 g. Only the intake of the fish-based dish had more than 350 kilocalories per 100 g. As an example, we calculated the amount of matoke with fresh beans required by a male child aged 1 year. Indeed, this is one of the most consumed dishes used during the complementary feeding period (Scarpa *et al.*, 2020, in submission). According to FAO guidelines, a 1-year-old boy needs 770 kcal/d^(49)^. Therefore, if this is all that is available, nearly 1000 g of matoke with fresh beans are required to satisfy his energy needs based on the recipes obtained locally. This is a large quantity of food for a 1-year-old boy, with insufficient nutritional content. Also, the Fe intake from vegetable foods (8 mg for 1 kg of matoke with beans) would be much lower than the standard requirement (50–100 mg for 1-year-old boy^(49)^) as well as the vitamin A content (14 mcg for 1 kg of matoke with beans compared to 400 mcg standard FAO requirement^(49)^). Conversely, if the meals consumed included fish or meat, the amount of food to be eaten would have been lower. For example, 100 g of a fish-based dish already contains half of the energies that a 1-year-old infant has to consume daily. The foods used for complementary feeding practices in Western countries are much higher in terms of energy density, even for vegetarian diets^(50)^. Therefore, further research is needed to explore infant and young child nutrition to provide clear guidance on complementary feeding and weaning practices among the south-western Ugandan Indigenous communities.

### The limitation of myfood24 use in south-western Uganda

We believe that the use of myfood24 with a local food database can ease the assessment of the food intake in less time and burden compared to the traditional interview-led approach. However, the level of literacy of Batwa and Bakiga is generally low, and accessibility to mobile phones or other computers is limited^(16,19)^; therefore, self-recording of foods is challenging. Local researchers may play an important role to collect the data on the myfood24 database for the participants who do not have a mobile phone. Further research would be needed to investigate the usability of myfood24 in extremely low-income settings, as this is the first database created for Indigenous populations.

Additionally, as the myfood24 system only functions with internet connection, more work is needed to make it available offline. This would facilitate the data collection in rural settings, avoiding the use of paper-based methods to record the data and reducing working hours and transcription errors.

## Conclusion

We developed the first online food composition database for an Indigenous population in an extremely low-income context. We used multiple fieldwork techniques, we engaged with the local communities and nutritionists, and we applied a novel framework to collect locally relevant information on foods and recipes. This database can be used in research to assess food intake of individuals’ vulnerable to malnutrition in south-western Uganda.

## Supplementary material

For supplementary material accompanying this paper visit https://doi.org/10.1017/S1368980021001397. click here to view supplementary material
